# Supplementary figures and images for: Size-correlated polymorphisms in phyllotaxis-like periodic and symmetric tentacle arrangements in hydrozoan Coryne uchidai
Source: Front Cell Dev Biol. 2023 Nov 22;11:1284904. doi: 10.3389/fcell.2023.1284904 (PMC10703359; doi:10.3389/fcell.2023.1284904)

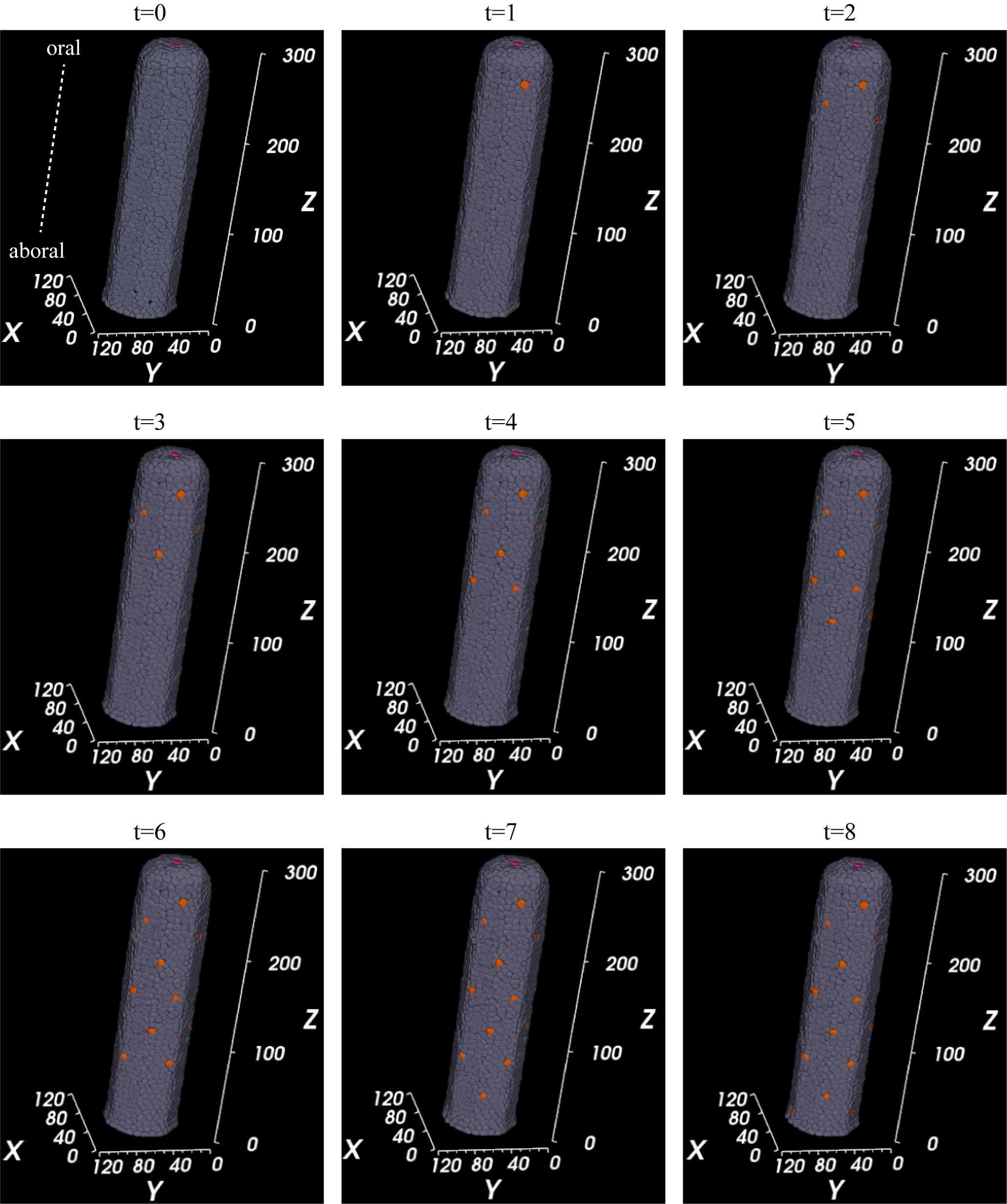

Supplement: Supplementary file 1 [file Image2.TIF]

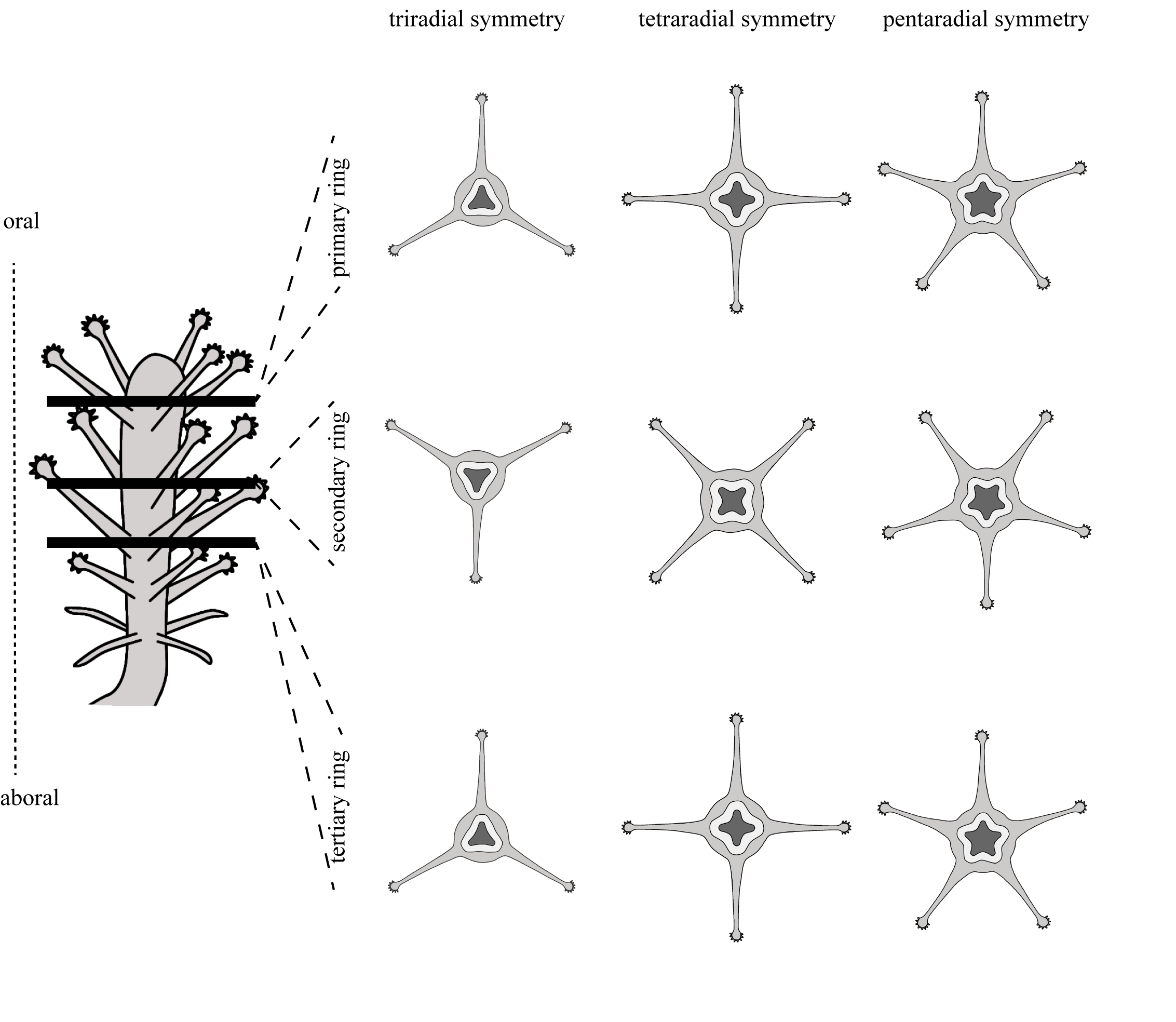

Supplement: Supplementary file 2 [file Image1.TIF]

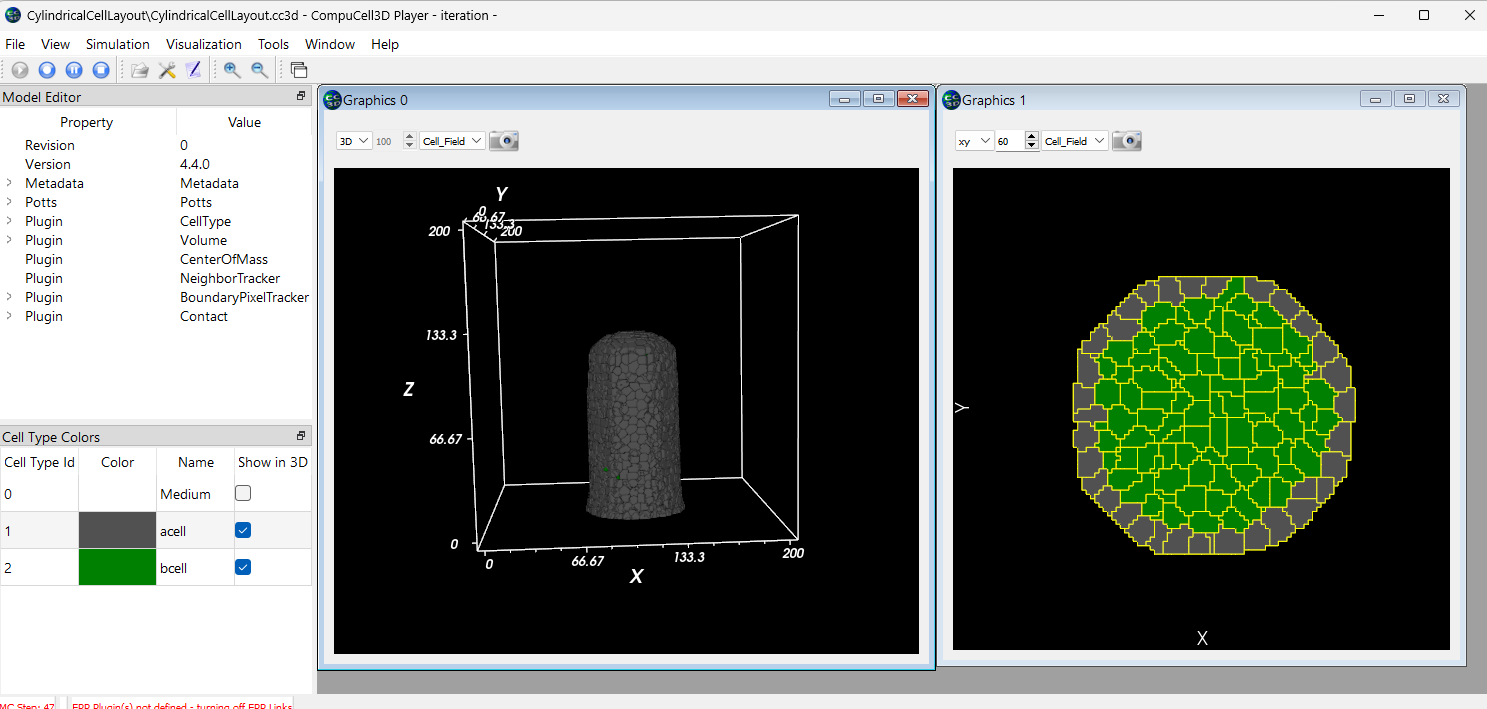

Supplement: Supplementary file 5 [file DataSheet2.ZIP › Coryne-tentacle arrangement/screenshot.png]
