# Supplementary material for: Size-correlated polymorphisms in phyllotaxis-like periodic and symmetric tentacle arrangements in hydrozoan Coryne uchidai
Source: Front Cell Dev Biol. 2023 Nov 22;11:1284904. doi: 10.3389/fcell.2023.1284904 (PMC10703359; doi:10.3389/fcell.2023.1284904)
Supplement: Supplementary file 3 [file DataSheet3.PDF]

1.76.3

80.61078918	188.9732309
84.31500582	317.3160801
97.91553444	65.26965206
163.6067392	255.1812455
168.5355854	8.615707389
176.9251134	126.4253418
211.4033037	72.87717948
221.9191451	202.5381647
229.3574596	303.3188421

-1

1.76.4

76.4548004	171.5526255
87.595865	281.1229964
88.99150729	62.25307325
116.1408476	358.100999
139.4060379	116.4380853
146.9873923	224.0108936
167.7286031	293.0638995
168.2079579	168.0966253
169.9502007	46.12938059

-1

1.76.5

90.0874326	342.0742111
92.41076239	164.4296466
93.74703145	66.91259783
94.85438265	253.3714219
172.3985284	109.9763675
175.7285751	196.8378321
180.8132915	22.21536377
187.3549897	286.6555737

-1

1.76.6

72.74102126	250.0277996
93.02293565	158.916812
94.50641511	58.81414936
94.79892175	327.5908082
134.5482386	106.1875113
136.4690508	216.0597626
147.2439123	8.994052951
152.1329744	289.8813433

-1

1.76.7	
85.27541332	270.1416395
88.59282145	145.5470412
100.9668016	36.47407618
148.9158978	221.9000842
152.1406391	330.6530915
157.661427	94.78078517
190.159589	268.5343594
195.0800889	147.4141606
207.4534155	36.23467488
-1	
1.76.8	
90.08586986	216.2743029
92.16456341	83.07431819
92.55732117	320.983205
140.9299553	155.4556432
154.0252842	20.3560236
157.5960663	271.1425417
180.2396916	102.4588878
186.2338668	201.8552414
198.2409562	322.1729457
-1	
1.77.1	
62.04075893	348.6890264
73.63225475	136.4807736
78.12102674	208.1481364
97.60013591	305.6181739
108.0187482	48.84018034
110.6927601	171.304392
115.9364267	352.4060362
131.3438757	228.8407161
137.0005351	136.562427
-1	
1.77.2	
80.68137902	130.9758546
92.5376179	207.7603504
94.19182335	37.31311059
96.42492512	305.5928985
126.8538001	165.8810467
135.064245	258.6178286
136.825193	349.1822979
137.2930196	79.56677873

−1
1.77.3

117.7964776	320.0374507
125.319472	69.3500197
131.4722485	167.2459765
145.8857629	242.6892769
210.8308415	13.44919269
223.1911087	119.3288854
225.6428295	293.1518575
230.4019252	196.2809343
259.7485409	250.3403764
262.6422472	337.8125783
267.0940242	54.07313938
267.2105492	142.6284632

−1
1.77.4

97.35363822	348.8789668
108.7879192	160.5091725
109.6882999	57.96424288
115.7949929	239.7505304
154.5627792	293.3788573
164.2192695	28.29253177
183.047336	103.4339716
186.3976226	340.82992
188.4078347	193.5046548
198.9382407	56.03910189
208.8366155	237.298897
224.9625336	274.2607994
226.1467819	23.69539673
236.9205008	158.5370973
252.8849853	84.33768115
261.0407007	219.4656813

−1
1.77.5

80.42256734	192.2940529
85.45597254	327.8654415
89.29258346	80.04368825
119.9250967	255.3868707
127.1180769	23.79930121
153.3619104	141.4705807
154.4088573	316.0187087
164.3532976	204.948591

169.9071055	71.87824464
-1	
1.77.7	
114.4588077	321.4925637
122.9576224	67.34027877
131.2967772	168.0893735
139.5492784	241.3020635
211.1162397	10.05479385
221.3939337	119.6857545
226.8001814	296.0438231
230.8643279	195.1660122
259.6209115	340.0434826
260.7196783	248.0819842
266.9476679	58.28000346
267.06383	145.8184662
-1	
1.77.8	
112.2142857	56.13892612
120.0778951	180.5745514
134.1133497	302.3764882
172.4019349	135.1560424
177.4541582	358.205209
201.0517005	229.6643581
214.6094037	44.30016025
234.4890656	177.5675626
237.5291068	312.4650524
263.9396367	107.7576798
268.5312409	2.247639781
275.8324956	233.233045
287.278579	322.2362759
287.9360804	174.1783889
294.7251562	39.43724546
-1	
1.77.9	
101.7565414	174.2270847
105.736871	259.5181666
107.0034255	349.1742647
111.0653891	75.16473369
160.463627	218.7586604
162.7443002	96.19790386
171.2346833	31.61243694
173.6338306.7711704	

187.4366978	159.7244477
188.1686076	262.456915
211.9367829	354.0498087
212.2962642	77.41700107
-1	
1.77.10	
105.1001508	359.8779785
117.0783368	273.5180652
121.3076509	86.92845033
122.5190816	173.2616006
194.575583	42.40077476
196.0903885	311.6015509
202.8215293	140.1507521
209.9455612	227.7001602
228.5667364	96.03647074
231.3538603	359.5207414
235.9400264	189.9813614
238.8783068	263.2254602
-1	
1.77.11	
117.4795401	132.5194789
118.4870603	271.7338344
135.648366	28.15684671
167.2455374	197.1814728
173.8472504	309.8773181
186.9627786	96.08590792
251.589288	14.05310376
253.5656082	130.5920082
256.9842186	272.4498442
286.5831757	93.50998193
294.246635	183.7158735
299.8997907	306.6670507
309.9005862	128.9227369
310.0113373	12.55881307
315.8241207	271.3920889
-1	
1.79.1	
101.5291724	34.46394714
110.3833323	267.5240733
112.9249637	160.7795879
151.285041	85.39005864
153.7729956	219.0354182

162.9587816	332.2890778
205.3825047	39.54021129
212.7130554	262.3488961
219.2606647	157.2676065
244.9308131	318.4773945
249.3512357	223.9341584
251.8426938	84.63563488
265.4425829	33.01838675
267.6058503	262.1856529
272.8101039	169.9504616
-1	
1.79.2	
104.6710995	327.8878239
112.9771472	213.2560368
116.2827567	84.66976385
148.3375949	276.3199802
155.3709066	35.49327222
163.7081925	147.3206896
194.5935443	335.1939335
228.9126178	209.2377867
236.6107045	97.70587939
261.840513	37.43748839
263.342904	270.9477101
283.4668834	146.1943369
289.7317405	332.8246132
293.4947027	225.7468709
303.1086537	86.70958917
-1	
1.79.3	
100.1778258	274.5284043
104.1153113	13.8544716
107.4415869	113.2393666
114.3203939	181.5045392
173.1086422	341.0709608
178.8088509	214.0249621
190.8630055	53.45141592
199.6551361	150.4308441
207.1457973	285.9246772
220.9868171	8.64245163
234.9838645	189.4261818
238.1040374	96.98572725
239.2510346	258.0503748

254.3314766	152.3416547
257.0278014	337.667262
257.5876851	30.30438374
-1	
1.79.4	
109.2181321	171.1915895
110.4804269	284.6704888
133.4020902	51.44358246
188.838175	223.4013176
207.5318825	103.7119426
212.860698	341.3481444
227.375459	165.9526828
244.7385352	276.1316457
255.1761653	42.87632966
264.5563577	219.1359005
267.5433588	118.3122664
286.2576027	340.3333629
-1	
1.79.5	
116.8598407	336.217488
120.8036031	218.3613509
124.2724607	110.0111756
151.8009183	38.67500943
182.9436896	164.9950796
183.2232317	281.031798
214.6500256	349.4773343
242.0186614	112.9074131
248.4892002	216.099528
284.5428958	293.5982596
295.897442	41.9185144
318.8998893	160.9004924
334.5460832	344.728784
352.7464779	220.2875418
378.7487083	106.7880446
380.1555169	288.7511041
385.8718709	18.20760002
420.8123854	175.9952466
-1	
1.86	
180.1347611	236.1470545
183.9075765	150.3400323
189.879689	49.79039076

196.5303329	318.003149
342.888942	97.05603692
355.3765941	358.3515473
360.7556523	269.0770033
384.5375232	186.7189649
538.3083173	50.1045591
560.4345691	306.8437473
563.6853117	218.6822397
616.7685026	136.0958923
742.1870502	0.1443189702
760.6984149	82.3890632
760.925891	270.5066176
798.113283	172.2224699
908.8174909	320.5067914
921.7455802	227.0517723
936.7298316	118.9430337
969.318988	37.92690653
-1	
1.80.2n	
136.144029	1.536799111
136.9258458	234.9920417
157.0414552	133.0109789
173.5610917	292.390056
188.8290822	71.64209405
217.2598642	177.9648675
247.0672769	14.04780032
282.5285998	241.2183888
298.6663139	113.9977363
328.8271374	309.7996496
369.0372779	176.0302746
374.5935903	56.54512811
405.7325006	5.302048558
420.1199952	252.5192644
432.5148929	112.5272078
468.4357165	58.72383596
468.46641	309.7190234
469.3547483	196.7559255
514.3750433	242.1908836
515.0001231	354.5942891
524.9826549	143.779732
-1	
1.2	

214.6661004	128.1876174
237.0393656	210.8154355
261.2109509	324.8619829
275.0036071	48.33710271
396.2021145	269.4810646
473.8998641	179.3186956
501.309964	76.6090603
531.6936324	9.042038749
613.0358782	204.4555365
668.3834081	305.6815836
858.3445981	252.7877069
864.9737755	101.4646766
888.0842321	39.14744817
982.117401	164.7543927
989.8895036	337.464601
1144.537597	65.05066178
1149.376719	286.4844987
1177.12407	212.3776295
1231.61212	1.052992851
1245.063388	127.894765
1283.132518	54.51752359
1312.373454	337.1154103
1318.318527	254.2304405
1320.957982	177.7371786
-1	
1.3	
192.3979504	355.2448974
194.1460167	66.27773978
206.2888539	278.848015
210.7628227	148.2232587
222.1154283	199.0902186
376.8054613	241.2678153
395.9017413	105.8575208
420.9374909	314.8094555
424.0222417	177.189513
429.5693556	26.62503886
715.1444917	56.02716238
720.0277912	271.6571882
749.5624645	349.3785773
765.6144218	131.6296968
804.9916828	209.9329594
1006.800524	303.1860938

1067.904756	174.7299355
1087.686235	18.17781774
1132.851845	93.99462545
1179.391297	240.2358619
1276.328013	330.5033637
1329.598079	144.7074893
1336.074684	48.81414364
1368.700502	231.2668509
-1	
1.13	
141.9276615	348.001507
196.5915272	66.91509193
200.3654537	154.0246256
207.5878658	244.7046844
342.8330491	211.4198404
379.374922	305.932919
418.3756934	31.74949633
425.5249743	129.0557325
590.5378612	255.4812932
633.5486178	193.9822316
685.3783654	10.17618837
729.1445484	124.0372882
877.2622759	353.0352002
880.8432466	267.8756838
928.9487011	192.084395
961.2436646	101.2241524
1067.296878	338.8417782
1101.631243	248.510452
1122.41119	55.36206534
1130.909662	169.3670946
1201.13227	220.6360444
1202.302226	314.3711104
1206.838446	116.5838396
1242.259384	27.75439512
-1	
1.14	
119.1046122	142.0802015
122.7694145	60.96604033
122.7729928	296.7813313
138.4747718	211.8692941
155.8291682	358.7723027
239.7857422	97.96234592

250.3603072	27.2302322
259.615581	178.9807835
288.8872958	270.9040955
330.2479964	342.8214597
369.5446794	141.6201121
384.3486913	219.8332389
395.6074271	73.31845011
430.823596	15.10105057
434.2209067	180.0715137
437.3384475	299.8589099
-1	
1.15.2	
195.9796222	71.54200015
210.1884553	269.3896501
211.2123881	177.1300403
216.4953211	347.1326183
345.712377	221.7675012
354.7484696	124.8417179
381.0861982	37.00800991
409.7610892	306.6963206
552.2132504	173.6725313
581.7252267	83.1473516
589.3063097	352.4063555
628.7549188	260.4235452
822.118377	41.82600364
865.0683087	132.4812497
867.7230888	220.8308536
930.3890493	306.0346012
1037.797649	74.12004789
1047.519162	178.3855845
1050.609827	2.849176318
1058.568616	256.906463
1106.117356	46.71056256
1108.412171	126.2690741
1120.99062	218.9938061
1128.969381	304.6473356
-1	
1.6	
190.9031189	166.1226112
195.0842482	236.5424108
201.5670505	83.95426886
208.6861386	324.7953108

210.7180051	23.68861644
338.9563147	265.1861767
351.8968457	138.3341501
388.0774386	203.3575825
410.7478648	351.3130725
436.3090131	59.2493229
620.6331329	303.2375226
665.1715348	227.395528
665.6117391	97.77793345
690.0788336	13.51029609
737.6777882	160.4038577
907.5010162	271.4780825
915.6622077	51.27242283
974.9735612	340.2275066
1043.625668	198.1318154
1079.267487	125.3986059
1186.512487	26.04691903
1218.379683	242.6510846
1223.230275	304.012371
1224.507206	72.68461075
1260.576003	157.0322271
1320.310998	217.6173025
-1	
1.16	
181.2497992	197.9335659
189.2624444	90.86092705
192.5411722	351.5308519
215.0481007	270.8607814
308.9456554	144.727882
377.5504607	34.06143449
413.6490606	240.2799417
477.9166435	313.9877051
491.0118203	187.3468593
495.5217361	92.51073308
654.6184851	275.3058423
700.0432335	133.702835
704.5932149	50.27069974
753.5653194	349.2940541
792.3646057	221.4809379
883.9988768	85.62979508
903.5169304	175.0972595
903.8025339	307.9689002

977.479806	21.91271974
988.2316096	116.4968916
990.0639683	256.3024796
1003.992542	205.5514511
1024.352016	64.08906874
1062.759825	327.3877726
−1	
1.17	
123.0360297	8.685430841
123.1165841	129.0054917
129.6615102	280.0548658
132.1441628	204.0405545
190.0322362	75.24158325
211.5708718	312.0769145
235.59998	182.9588258
238.6471702	239.1619583
252.8805993	23.52103265
264.4664521	127.0376831
295.9582116	76.52226
303.4189207	276.1169726
323.2784902	320.3911572
327.2113152	207.2740965
328.7359654	141.8400287
334.2797862	26.52907779
344.5616072	90.46870019
356.0511959	266.1584038
−1	
1.18	
173.0529086	351.5246268
188.0819611	84.3348623
198.3469166	269.2487168
200.4886836	196.5829265
313.1192381	149.8932002
372.1709441	29.94447496
414.9277629	231.9165939
465.8942245	314.1242582
489.8185159	97.21222292
503.2974828	182.477478
647.4788949	272.8041354
699.4918835	140.9999572
702.5116991	50.78597705
743.3527908	354.5191812

785.2727199	219.7670967
898.6230929	85.79137979
903.8188384	308.6539398
906.0378567	171.7920722
974.8433795	22.97387593
986.704953	123.7937789
989.4369451	252.1400364
1008.616935	202.0069901
1032.683375	55.19979499
1049.054333	327.7617932
-1	
1.19	
186.5210607	165.1055482
193.2492595	5.357611552
193.2755453	281.5066343
213.4810019	90.47127365
309.2545577	199.9097837
377.0975551	336.9536064
407.0067946	134.1893061
456.5914282	23.80393753
462.5306784	173.8855697
481.5946742	258.1447103
592.3650475	72.44973236
625.2379323	210.8964727
646.428917	316.5658338
684.2360577	1.160690763
731.9643723	143.5210032
821.4437045	273.0375971
841.575087	181.5308994
846.7095943	34.80426896
920.3759242	344.4145848
923.2770487	106.3849819
927.4785554	220.5424207
967.3051524	158.3579344
975.7794492	25.18583088
980.4849224	315.8357158
-1	
1.22	
165.8983492	288.5623368
175.0950028	30.41244877
178.5175652	165.1842783
246.7536424	233.755594

252.6312087	328.2565015
258.0865022	97.72831437
320.2363617	163.4183462
325.463582	30.29187487
369.3251735	275.4763751
457.0325295	208.9778055
461.0089978	99.20287256
489.1643861	335.3094507
605.408709	162.0893209
620.5041709	49.89847704
634.6866031	277.5899958
773.1426673	352.7531225
777.3845488	114.1646876
780.2266143	239.5547022
898.9372387	297.3541884
917.7645316	176.848642
929.3142301	55.63644776
972.5824702	0.6631749146
973.5113634	242.2118532
1012.95361	122.2027597
-1	
1.23	
213.5712332	207.6240454
224.1789662	150.974872
225.4735438	71.36986436
232.4032365	358.0767381
253.1546577	286.6668728
396.8323453	356.2218689
437.6047464	181.7058836
452.0923612	112.0636452
518.8614235	43.83192596
524.6234251	241.8742207
576.8424263	311.8532173
700.3820325	142.8886736
745.2469716	12.5451453
778.4704482	201.0532513
809.5963896	274.6245075
829.2536261	69.53437879
917.0236774	339.8182784
993.4825804	161.8462767
1015.520423	26.36040963
1057.393517	212.2922349

1090.134087	103.9993092
1134.270183	302.6655814
1216.388307	348.4706462
1231.743024	174.6088825
1265.336772	225.4821363
1281.460755	40.45794622
1302.664306	125.7874007
1346.380777	306.6478662
1362.742498	190.0336244
1395.001815	8.871949154
-1	
1.24	
134.9910152	203.5603861
158.5693102	310.3501543
163.1505884	116.0331541
175.6637156	26.52152642
278.5292283	254.5153315
292.3439286	167.4393698
333.5568999	59.67807612
343.2953872	345.0633845
428.8896858	210.4550035
457.3392382	131.7073592
476.9876814	23.15315944
499.7895556	292.6820096
569.464048	178.1426123
579.2876521	89.96027697
604.4653241	340.5256946
619.3762692	249.3079733
626.5924542	33.27695468
648.4563633	150.3622232
676.6267036	302.0910172
678.0647911	202.0330817
-1	
1.25	
171.670018	309.5371047
207.0183075	172.3987171
218.177263	41.70684638
287.9994266	225.5189426
305.9953758	108.702703
333.8177522	6.036402218
455.1519257	172.5155851
469.0305411	294.3929003

524.6513261	47.5972423
613.9831247	113.7838242
623.4539978	225.5877151
641.5072842	359.1903787
762.2334966	179.8087354
799.9883384	39.08099032
804.1136091	299.6146811
985.3190518	88.02253857
1002.063553	341.9505968
1022.146073	145.9954048
1081.272497	245.318827
1258.371775	18.77589405
1300.768502	210.1051359
1375.45974	309.0043695
1460.861279	63.2848441
1523.528354	186.1349807
1577.739331	359.0368693
1620.185088	252.9700194
1620.671843	133.3760985
1646.784888	316.2745497
1677.884928	57.21488373
1689.315581	205.0673439
-1	
1.27	
181.7235066	116.1432967
194.3405727	208.4904043
200.4242884	38.60145236
204.9473788	305.8812389
291.8314805	256.5202953
385.9751711	162.0341005
389.8472662	1.439967719
397.3132575	69.15063315
489.0073294	218.2185824
490.3020314	286.8325222
622.39375	114.0858728
636.6426803	27.96287593
701.3618813	249.6051279
725.7831526	329.1657183
734.5367229	177.0110442
735.1163576	59.47002641
873.0561726	130.4242179
875.530366	220.5482878

881.1378771	76.74868769
887.8801122	282.1974888
905.430903	26.20392469
954.3167356	178.2737168
982.0437961	328.6212511
1003.247203	248.492888
1016.253409	55.50454844
1022.65532	112.7515628
1023.285452	297.2438967
1024.804006	9.549019276
-1	
1.28	
198.0623069	99.7875786
206.1391123	267.5121381
212.1299406	183.4308206
219.0353815	4.613437628
361.786347	318.8006172
376.7696032	50.80628861
413.9058927	222.9283288
423.7897753	143.1196435
532.373139	8.129196643
572.7912523	274.0772537
616.27156	174.1572455
634.2379929	87.42590018
729.2264037	323.2158284
787.1343575	231.9914716
804.583832	25.27876176
862.7408952	132.274859
977.6892704	268.7377145
991.8433832	184.247952
1017.146054	83.3678374
1031.27143	346.3037701
1055.74221	231.5618816
1121.540752	128.0152558
1158.109703	298.9179707
1176.216417	41.48105862
1189.554681	192.840812
1203.258017	257.2262501
1214.499453	355.7904033
1232.861367	77.63754029
-1	
1.30.1	

194.0991736	204.2720853
208.0227046	97.70989865
215.4471114	292.1089193
216.2189192	13.71523259
315.8747505	160.452071
471.2847452	244.2987413
475.5488268	342.0272749
485.9707784	64.27229999
597.7828584	123.5721364
628.3101164	293.6861879
649.4883282	190.2409929
721.226391	20.45412597
806.8345966	85.25924558
845.2363442	256.6557036
853.5125871	329.4307831
881.2158053	149.2329248
933.8553771	32.16422115
945.9192611	286.0258674
956.993334	204.2930383
990.3839602	152.5990178
993.7424204	93.9103763
993.8744326	253.8518107
999.5117548	342.7439909
-1	
1.30.2	
202.027708	40.91022892
217.8413976	134.3012617
223.3953716	315.0874355
241.9398343	223.9860585
430.7482159	80.74846106
450.3178544	353.1472934
497.7208878	254.6635638
532.1218878	179.0816241
728.2656092	119.4296029
772.7621388	303.7983863
808.6866297	23.12091696
911.5818646	216.1868409
1040.853415	74.76599198
1108.813525	346.5800911
1120.0526	161.0468874
1224.949343	272.740788
1358.207378	47.56430793

1398.003682	219.9410733
1439.490738	118.0889843
1520.202891	338.9583351
1579.293996	174.9435886
1613.50774	79.45725041
1659.156327	122.4222634
1659.450079	13.80844457
1662.513469	281.6275377
1671.82906	212.8800936
1716.922342	44.44910773
1751.978592	325.4314748
-1	
1.31	
203.5891987	92.92597697
209.6346576	274.9929458
218.2308854	356.7336674
234.943686	182.1138639
360.450314	39.09061427
404.3354462	225.2077991
442.5769038	146.1252449
465.3642528	312.7267993
644.1010927	84.55993425
655.4374068	358.1991539
719.5778962	178.1754498
759.2255662	250.2589341
883.7443645	26.3450723
935.8756609	131.4753467
964.5537569	309.1573473
1001.544653	204.4847256
1109.337326	71.40197912
1165.076192	166.8240659
1173.018943	346.932216
1192.049242	255.5296653
1250.993603	113.3983338
1252.645007	198.036268
1255.216795	22.46042154
1299.899795	304.4751002
1331.279187	142.7238208
1338.3037	60.03285078
1350.249082	341.3462322
1367.034769	242.2954933
-1	

1.33

251.3247643	320.9860399
265.7891414	52.04964138
274.2139882	227.3171352
294.1866052	140.8869546
451.3604625	274.2963231
567.6791646	181.6085305
567.8930934	101.4344101
570.3109376	357.5403189
763.9343403	44.9158703
855.4717672	231.9981719
900.4779507	307.1521215
917.0773391	147.8422855
1089.70625	18.33302093
1137.614959	82.00089364
1197.008235	266.8776953
1248.888957	196.3089356
1422.782381	134.701726
1445.801794	327.428006
1551.871387	229.5606565
1563.575182	45.03613444
1684.064606	281.838515
1685.478988	169.3029807
1695.221423	88.69017644
1738.539297	351.5000477
1769.254801	125.0985806
1794.352139	318.0305097
1798.700724	51.42782711
1806.317188	204.0992606
1813.723946	251.7989176

-1

1.35

172.5602644	0.05724691675
173.9298897	102.6487236
179.2475725	205.4676757
193.3293464	287.0201653
294.5415528	52.74667512
306.9333605	152.7786514
387.8606396	345.6522637
391.5945847	227.6802908
447.8922432	96.20632993
502.2365765	289.4437331

550.3122444	36.78618564
556.0270009	172.9178764
666.8030208	236.5396588
671.9036689	348.4027301
672.920274	111.0719685
740.7600287	294.5785105
764.2657755	54.68886542
788.8711368	174.5176084
826.0589038	356.8704243
826.0819692	120.0465905
843.6993228	236.9988792
-1	
1.36	
152.3377996	59.37758268
153.2453541	151.1455959
154.9939927	242.6966182
159.2063639	336.928507
276.1220004	291.0511242
279.4538773	102.5690769
298.6722665	188.8063693
302.6168313	24.73223745
391.2369962	239.7748441
455.6613055	141.9738864
462.7841994	332.1230819
480.034464	62.72859644
554.7626014	277.6762916
575.6264273	198.7470304
589.3638285	15.71905544
615.5539883	114.7619583
629.3882776	320.8144737
669.8751624	227.4595853
679.4048741	63.41511924
703.4912815	158.0173438
-1	
1.38	
138.3564989	190.1510996
142.0882552	1.269125143
144.1065138	276.224269
158.5263193	99.2681918
274.8178313	143.4570319
276.738884	49.86864451
288.0321912	233.2706463

295.4852551	319.3075641
406.5276505	97.27267648
412.8175164	15.10634238
421.850415	181.9984768
444.1817476	268.7525408
485.167619	321.4379626
505.9681769	69.19644118
507.1651332	136.0387199
518.0665277	222.7888053
523.1838849	14.08460958
556.3019142	174.256799
556.7888623	283.7612985
564.2185861	105.5687167
-1	
1.39.2	
219.7091907	311.7723844
220.5434215	13.899233
251.7814185	93.31633562
259.641742	173.1512647
259.6880015	235.8932513
445.8201611	150.0260379
452.0186524	275.6397188
487.2859561	346.5615167
490.111318	59.64614201
506.6039387	206.672145
669.7358042	113.0357893
703.4881581	29.85513948
727.0040191	319.2060855
762.2158688	173.5979791
764.4204496	249.8706352
907.4044382	61.16838441
948.298192	212.5758642
956.2265377	1.521730205
976.6520523	132.2962206
989.3606219	302.1615874
1111.58899	255.3079136
1113.16703	48.06181039
1114.248782	183.9138944
1122.254784	343.4215059
1171.553783	118.7560553
1188.507649	299.7081768
1211.678469	165.6722315

1212.359195	28.32210959
−1	
1.37	
165.4617286	183.9825573
174.1831397	97.24216055
188.737198	305.30224
209.513012	5.919055786
288.1478049	233.9849824
327.0000887	335.6284346
330.4744843	149.9158114
380.3738631	53.14928768
433.548829	279.1196299
462.8611778	190.1612919
466.3059016	4.90237167
533.1774617	118.0734178
601.0779316	339.3085375
613.8497163	215.462412
639.0261528	38.1658593
680.7615755	74.23692082
727.5729569	292.4277864
760.663763	1.483292931
778.7844053	111.209809
780.8498668	195.9038632
806.7495199	342.9186331
824.8011011	232.2011371
848.5346206	34.28323721
854.1904945	153.0577883
−1	
1.40	
166.0851007	180.6070535
169.7070203	279.9142173
178.2396428	32.54740433
223.8027838	105.8781306
290.7567656	232.5394895
306.9106629	331.8307215
384.1296688	164.5127576
390.8204399	54.97576932
433.4634015	277.4555932
499.9848673	108.0438231
537.2743916	215.5496457
555.6792041	350.6024249
661.1681809	292.4170867

666.2351801	52.0358525
667.6585543	162.3506764
738.4928387	111.6571138
757.7589858	237.9792994
773.862175	356.1194152
818.9708557	176.5296897
820.2917233	300.8314759
842.5054891	58.57799852
-1	
1.41	
215.1993061	23.8596982
251.2142619	96.08250951
251.5845176	163.732628
254.0216407	235.1949807
258.4660936	308.9365248
453.2966248	352.6657308
463.2597067	57.20291155
518.0230194	126.5470342
545.2680762	281.244683
545.9953837	201.8313777
762.0686466	329.7027094
792.1629777	34.44788881
837.0211604	162.5609499
837.4393581	98.20769237
840.7194297	248.2926578
1050.39829	4.984467219
1057.545584	291.5217255
1119.10821	147.8098053
1139.722618	77.01741781
1157.285979	214.4475005
1226.42107	332.8762506
1284.073749	266.7224629
1291.250913	28.61787014
1324.554536	112.7479271
1346.651091	179.1107669
1370.452847	301.5480952
1378.090389	230.8756169
1401.508946	71.55171318
-1	
1.43	
132.7945379	145.3052243
135.451834	321.3257822

152.1230422	57.84380882
153.4618531	224.325443
277.9552335	280.6600025
279.6764147	1.715103991
289.6524048	105.3934713
331.7636507	193.9923328
367.9335216	55.55910713
397.0681786	318.5652265
401.0245878	140.7009839
406.7667994	243.6222472
423.8735569	195.9772129
445.5562138	84.83458343
446.4681648	19.55417063
453.5210108	279.5177218
-1	
1.45	
206.3561965	94.74792896
224.0504523	182.3579732
226.8863998	354.5325192
252.3547668	258.1628639
454.9490704	54.53634267
482.2909475	222.3761461
526.6499707	300.9509374
574.6957514	136.4824876
728.4018627	179.2232662
744.6368156	356.6628209
814.6273047	67.97919531
816.436277	253.7299263
1081.662139	306.3866452
1098.093299	127.5834836
1131.490015	205.1647728
1164.588774	24.41940339
1192.293076	78.3739355
1339.988133	343.3804416
1348.440318	262.7056512
1354.517623	164.7422907
1364.332423	78.67754714
1395.701049	295.6924222
1397.615098	208.5038918
1429.552449	120.9525939
1473.4679	28.84218076
1507.847806	338.2292605

−1
1.46

185.1225453	196.2550911
198.7063409	6.940368937
209.0946428	121.3400757
213.3744293	279.7688398
321.8630074	66.6480738
407.5944818	156.1070783
421.9841199	321.4827206
460.5071501	241.428314
492.888276	18.31948404
547.4795483	109.2209233
695.1251181	196.2550911
698.7584379	62.0420867
729.6945262	280.7862753
807.9912453	155.1907476
814.3340199	345.7954411
898.735896	103.8609531
931.1478486	34.74337655
933.8531528	247.6689477
1007.401255	303.8219765
1020.776148	191.3959413
1036.118404	76.68154693
1045.125361	355.5443014
1062.894899	134.7122097
1082.382319	255.4145007

−1
1.47

235.6974957	63.6712239
248.2356483	286.8247778
248.3460602	149.3199625
277.1112862	353.1906392
291.5383548	211.9850854
461.73904	118.3986289
547.4189948	177.5977877
548.1552962	33.18884384
564.8926999	319.803247
646.2925775	247.1596455
879.2636138	29.47934053
882.0781948	123.3409305
939.6329624	334.2937608
985.8811641	210.2441038

1082.474412	278.1014272
1206.217687	134.5638094
-1	
1.48	
224.1134888	245.1500384
226.0048895	314.4837389
228.9370717	18.00401464
237.6436529	162.6290128
253.3587146	101.2221055
426.3797068	203.4775597
428.0338917	346.0304183
440.5930472	271.1902355
490.6063752	63.65616889
522.1703952	129.1071186
724.3165163	232.8610522
726.6652004	319.635313
810.223616	23.04931237
811.1160682	97.82672952
816.7451245	172.5643025
983.6586961	279.2709838
1033.092417	127.7988414
1053.735527	59.27275276
1060.105699	335.6881268
1088.53218	205.3905667
1165.430107	139.1617341
1167.14246	262.2733929
1181.221247	6.405789608
1202.529449	73.44007895
1236.535606	206.3583458
-1	
1.52	
210.0073722	174.9779572
242.0818044	105.3724858
245.52731	259.0798381
284.984739	338.9618307
291.821286	38.17200803
397.9704256	217.706633
439.1641443	126.0315951
445.6519263	60.81465365
454.3770858	303.1559416
574.0830748	3.97819648
585.3156901	166.8510529

621.9125421	250.3962391
699.1276314	86.97825179
758.0228975	199.4411409
787.3236724	316.7998906
849.9461827	28.64203128
858.5845107	138.5359037
926.9272047	231.6280917
1016.049628	181.4835734
1017.556337	86.29175932
1037.840403	348.5980284
1076.597108	280.9381809
1110.270435	213.83546
1131.125618	144.5829542
1176.114391	37.90887201
1211.305719	249.8533516
1218.494392	178.9781988
1237.020104	324.678571
1255.240657	80.80955084
1342.753356	12.43426813
-1	
1.54	
106.7049988	238.4073085
130.6037561	118.8207637
135.0739572	351.3097119
153.15467	182.6994188
184.6902545	36.09479657
188.1576546	304.9424858
222.601473	226.0522406
229.6716519	130.5222267
248.5124381	175.5875246
258.3798045	351.2734811
267.2873713	51.33631673
270.2090986	296.6937197
-1	
1.55	
102.8300414	249.9690752
117.8117018	70.28249054
123.3893571	338.1003288
133.0210319	167.1035167
185.5238449	25.48149846
194.0979967	294.3655544
197.1399974	224.0567791

197.4409728	124.5867822
227.3434765	340.6162624
233.6078081	170.4667489
235.5427845	79.48162401
237.0292328	247.1650493
258.0119671	21.88753013
262.2803125	155.5885785
263.5378882	310.1250832
-1	
1.56	
94.40994601	295.1307709
99.48867167	207.8114795
105.8846647	30.73824613
116.1942131	118.6191147
176.1651771	86.4726005
178.0471717	261.6534373
180.3370394	339.819795
181.3076665	220.8155388
182.051644	140.0661853
194.7474368	18.3208586
195.741911	297.0850213
-1	
1.58	
245.0975859	203.8922958
267.1670412	6.199703715
277.5214353	282.0380357
283.2757353	137.3633158
297.0612984	82.68654439
516.1416407	244.5166252
534.1626362	161.8837937
546.934151	338.5896939
563.1523853	43.12325982
671.5566818	288.5704242
702.9315419	109.2366958
739.2885678	200.0961283
827.6757902	15.00590561
899.0982693	244.7231061
915.7785185	152.4622244
973.9667532	73.70866739
982.0991202	325.9397696
1086.442008	193.372971
1101.221034	124.0348621

1118.071807	32.03047391
1121.296865	283.2492323
1143.875857	235.5945236
1186.610292	159.9215269
1209.272471	87.59674258
1212.287571	351.5094777
1213.539385	308.8812635
1247.460501	44.16036475
-1	
1.59	
115.6947352	253.0775521
117.9881635	139.2812085
134.2565378	343.617188
137.6439744	65.94343679
190.8923896	184.8031959
210.0260063	100.6380757
218.9605331	302.5029043
243.6341223	19.73414836
245.3782887	141.8990551
258.9131143	247.887999
270.2189046	85.66720678
280.4246977	185.4896325
285.6101611	322.0666399
294.4986758	18.28700401
-1	
1.60.3	
148.3250835	176.6068526
158.8574206	327.8332207
162.8498988	81.24047917
172.4571121	18.58332178
173.5372346	110.9864479
178.5271019	242.853152
303.151065	145.2941951
362.2284235	35.48106755
377.5932888	216.0818682
406.4818651	335.8514788
517.953822	271.6080214
538.4043736	95.67205131
617.0143241	171.7193837
722.940081	5.667635549
784.6415177	55.22636105
852.2849362	215.8511597

920.6954923	138.710194
933.4847733	317.3012697
1099.003916	5.671302165
1106.409565	185.8606577
1114.294438	84.99976393
1154.178622	264.1352824
1237.043809	137.1487111
1251.499929	42.68545872
1269.952447	224.2077972
1278.137248	310.3590586
-1	
1.62.1	
120.3945084	317.8391762
121.1577448	241.1189896
124.1378785	54.79015031
124.5678868	161.583945
181.826082	110.1245202
183.1252057	2.010998083
185.30722	204.4376358
195.5229301	279.0594231
220.1434609	58.03378761
235.6087842	145.349903
249.0529534	338.9705215
250.1591478	238.550829
270.7538571	78.21796641
275.8154148	175.337895
281.5561713	293.1459415
-1	
1.62.3	
149.0798159	249.3021939
161.8357304	33.56245188
162.7158074	132.7476828
164.9779241	308.9226226
216.7642756	202.9897148
226.6602252	82.34113275
262.9050413	343.8839081
297.1621473	271.1702229
299.7161108	149.5464542
349.8765143	103.7824002
374.8996553	42.54475895
416.6687686	214.4991515
421.07406	314.4744692

474.3134393	138.1235092
486.3009311	35.1191257
488.2742596	263.5629765
501.1260327	94.25036034
511.707477	332.2100742
532.112551	184.907865
545.0161983	253.4172612
560.5959034	33.31499121
568.6066862	121.2421741
−1	
1.63	
213.4874263	206.9167714
231.891027	340.8436209
238.1387419	128.3582698
238.8712886	268.2658489
243.4440008	57.40207042
407.8065875	11.66527118
410.5323639	168.3722891
422.6163208	300.052766
439.4645482	242.5963762
447.8995312	82.98886508
664.7655806	204.4849282
672.05187	274.0095121
682.6561578	343.5797334
704.2549231	127.9549425
742.4754392	44.54757554
865.6798841	246.0309564
874.8816459	77.95139336
883.971538	164.1563201
921.8650274	309.3464273
970.3920137	26.55845676
992.5824125	205.9448949
1017.977533	108.376296
1028.06022	271.3736864
1051.968697	343.9314163
1052.709166	66.66689667
1084.520877	160.6745898
−1	
1.66	
120.023309	338.8639602
120.2240778	202.4708519
132.2349276	72.57743984

181.077544	8.135694956
190.5331516	267.1878704
195.8571008	151.3869496
210.2904453	328.0410191
212.1210194	76.8301428
220.4426701	195.6621147
239.1526259	10.85378576
243.191788	272.6200919
255.1069014	149.3541191
-1	
1.68	
117.253427	31.49108077
126.6130203	313.7704397
127.7786955	127.4067015
131.9485215	204.1353968
169.3715666	356.0278268
170.2668537	261.0430438
172.5094151	73.87263748
176.2330636	164.1202722
237.2934765	209.6696417
238.6183525	316.3703156
247.6263375	113.2322345
252.7082197	27.22833049
292.7141371	257.3977031
301.5970187	349.4080969
305.6372972	169.4313826
317.2823342	73.98594106
-1	
1.69	
121.2945701	100.2767554
123.2210063	11.73032764
130.3437014	189.2419121
133.1718027	288.019427
220.8619454	60.51111392
227.5867624	332.8590977
235.9524603	236.0628455
244.8051569	149.4161834
298.4832749	191.7664805
300.490569	288.6811678
314.9235031	102.5825408
322.6706029	8.199981271
353.3161646	240.0197185

367.0959835	353.3358083
367.2361641	63.61528023
369.1470241	155.6225138
-1	
1.70	
197.5581908	21.04401567
199.735828	165.5976493
208.7599127	223.9760568
210.5289933	307.6411689
215.2065824	95.59965205
368.2893395	344.139299
372.025307	53.3935214
388.1271342	195.4922817
394.8917837	136.8755544
409.9658747	268.9849863
609.4637097	19.39380124
610.5728491	319.4692329
610.5856639	95.43964771
632.7974535	219.1770025
684.3690088	161.0655617
746.9605764	268.9850267
805.3481605	43.04491555
807.6425781	343.3906201
857.4432396	126.4113043
879.9362044	194.691516
893.3611987	9.258753068
894.4919328	272.6698862
924.9342691	80.75575777
931.432099	220.0677351
946.9842551	158.7302613
-1	
1.74	
138.7137522	189.8363557
139.8886791	96.40212317
141.7554374	5.297719408
149.6370875	276.5993079
229.886423	322.0534573
237.4457504	59.23814628
255.0137725	227.9038854
284.455769	140.7482032
305.9395085	17.16810772
320.252745	275.1954394

347.4303628	92.54071648
367.6738633	185.058913
381.0238213	323.6342409
411.1840652	46.24186425
430.1871333	235.2796513
448.5481748	5.506306989
461.7242493	288.4041207
463.1718134	132.9275816
491.172759	80.73555821
497.595358	183.5278817
-1	
1.75	
137.5061585	94.71170691
140.7036397	209.0221611
143.7698264	328.0501855
201.1666236	38.08831273
216.1563869	140.3534626
248.0549141	276.3839149
267.9228375	82.2561569
311.1871927	346.1166234
318.1580265	212.0742087
373.9793463	157.9242694
389.2521847	37.3380447
397.2499215	281.2371332
429.7529834	97.67190526
463.5616653	216.5587889
467.1362594	339.6469177
494.4715693	51.50301393
515.3920244	154.6367192
521.8166305	103.911694
530.0660955	272.8508183
536.688016	355.2485184
567.4611816	219.8777961
-1	
1.81	
130.4123754	359.5330282
137.6980171	245.2273449
142.1194021	136.2919649
153.326001	58.5350486
222.4432233	303.7225052
236.9448386	183.3988845
261.7481354	80.73810004

297.5432868	7.719664269
300.9617394	245.5150334
334.3362325	140.7153806
389.9624604	51.73312114
391.6607554	195.5975684
391.9251737	327.1798721
439.6656161	6.920365038
442.2775464	96.86652602
444.3060079	273.7786572
463.6881907	158.2530834
491.4191289	62.6875619
492.3542879	319.2046476
505.1362467	222.6298063
-1	
1.81.2	
136.8377276	197.5670512
141.3520814	343.8617444
146.5574335	86.89466163
181.131062	260.5232662
210.7025274	21.15244951
211.8148348	140.1138668
262.3184594	197.4899026
269.485221	331.8523098
293.8171472	85.86325669
342.9359131	262.9033511
345.8710304	142.5782817
367.6036392	18.84255537
400.6705325	201.101335
416.2028681	82.81050932
433.9176577	323.3902651
464.7189332	140.0030552
474.7395961	19.76749941
485.5270329	264.1020026
505.8642987	75.90413632
520.6937316	196.6827991
547.3827189	328.1245759
-1	
