# Supplementary material for: Size-correlated polymorphisms in phyllotaxis-like periodic and symmetric tentacle arrangements in hydrozoan Coryne uchidai
Source: Front Cell Dev Biol. 2023 Nov 22;11:1284904. doi: 10.3389/fcell.2023.1284904 (PMC10703359; doi:10.3389/fcell.2023.1284904)
Supplement: Supplementary file 4 [file DataSheet1.PDF]

parameter	value
D_a	0.7
D_b	0.6
D_c	0.8
s_a	3000
s_b	1000
s_c	1
k_a	0.11
k_b	10^{-24}
k_c	0.1
dt	0.1
dx	1
dy	1
T_a	10^{-9}
T_i	10^{-7}
T_{ti}	$3x10^{-6}$
